# Ghrelin Exerts Analgesic Effects through Modulation of IL-10 and TGF-β Levels in a Rat Model of Inflammatory Pain

**DOI:** 10.18869/acadpub.ibj.21.2.114

**Published:** 2017-03

**Authors:** Faranak Azizzadeh, Javad Mahmoodi, Saeed Sadigh-Eteghad, Fereshteh Farajdokht, Gisou Mohaddes

**Affiliations:** 1 Drug Applied Research Center of Tabriz University of Medical Sciences, Tabriz, Iran; 2 Neuroscience Research Center of Tabriz University of Medical Sciences, Tabriz, Iran

**Keywords:** Pain, Ghrelin, IL-10, TGF-β, Formalin test

## Abstract

**Background:**

Ghrelin is a peptide with attenuating effect on inflammatory pain. Both anti- and pro-inflammatory mediators have a role in the nociception and development of pain and hyperalgesia. IL-10 and TGF-β are anti-inflammatory cytokines and inhibit the expression of pro-inflammatory cytokines related to peripheral and central inflammatory pain. In this study, the effects of i.p. injection of ghrelin on the early and the late phases of pain, as well as serum levels of IL-10 and TGF-β, as anti-inflammatory cytokines, were investigated in formalin-induced pain in male rats.

**Methods:**

Adult male Wistar rats (n=48) were randomly divided into six groups: control, formalin+saline, ghrelin (40, 80, and 160 μg/kg), and morphine. Ghrelin was administered i.p. 30 min before inducing pain by formalin. Pain induced by intraplantar (i.pl.) injection of 50 µl formalin 5%, and pain behavior was studied for 60 min. Serum IL-10 and TGF-β levels were assessed by ELISA method.

**Results:**

The findings of the present study showed that ghrelin with high doses (80 and 160 μg/kg) significantly reduced pain intensity in both the early and the late phases of pain. The serum levels of cytokines, IL-10, and TGF-β1 showed a significant elevation with ghrelin at the dose of 160 μg/kg.

**Conclusion:**

Ghrelin is effective in reducing the intensity of both the early and the late phases of inflammatory pain. It seems that ghrelin exerts its analgesic effects in part by increasing the serum levels of anti-inflammatory cytokines.

## INTRODUCTION

Inflammatory mediators enhance the excitability of nociceptors in order to protect the injured area by increasing pain sensitivity[1]. It has been proposed that the restoration of the balance between pro- and anti-inflammatory mechanisms may manage the development of pain[2]. Numerous inflammatory mediators act to generate and maintain the development of pain and hyperalgesia[3].

Cytokines with more than 100 members, referred to as a group of multifunctional substances, have effects on many steps of the inflammatory state[4,5]. They are produced in a cascade model and affect the activation, differentiation, and proliferation of immune cells. Cytokines also control the production and the activity of other pro-inflammatory (such as IL-1, IL-2, IL-6, IL-7, and TNF-α) and anti-inflammatory cytokines (such as IL-4, IL-10, IL-13, and TGF-β)[6]. Among cytokines, IL-10 and TGF-β have potent anti-inflammatory effects, repressing the expression of inflammatory cytokines such as IL-1, IL-2, IL-6, and TNF by suppressing macrophages[7].

Pain and immune system have mutual impacts on each other, which makes it hard to conclude that whether the pain reduction is the cause of the decreasing level of pro-inflammatory cytokines or the adversely decreased production of pro-inflammatory cytokines results in pain attenuation[8]. Sommera and Kress[3] have indicated that pro-inflammatory cytokines induce or aggravate inflammation and neuropathic pain[3], and the blockade of their production or release can be an effective strategy in treatment of pathologic pain.

Ghrelin is a 28-aa gastric-derived hormone and acts as a growth hormone secretagogue receptor ligand. It is released from several peripheral tissues and central nervous systems such as hypothalamus and hypophysis[9]. Ghrelin receptors are found in different areas of central nervous system, especially in hypothalamus, pons, and medulla, as well as in areas that are responsible for controlling the pain transmission[10]. Ghrelin has also inhibitory effect on inflammatory pain through interfering with central opioid system[11,12]. In addition to analgesic activity, ghrelin has been indicated to be a powerful anti-inflammatory intermediate and inhibits the production of pro-inflammatory cytokines such as IL-1β, IL-6, and TNF-α by activated T cells, monocytes, and dendritic cells, which reduce pro-inflammatory responses[13,14].

In spite of the proven effect of ghrelin on anti-inflammatory cytokines[14], it seems that its effect on IL-10 and TGF-β in formalin-induced pain has not been studied. Therefore, in the present study, we have investigated the effect of i.p. injection of ghrelin on the early and the late phases of pain, as well as on the serum levels of IL-10 and TGF-β, as anti-inflammatory cytokines, in formalin-induced pain in adult male rats.

## MATERIAL AND METHODS

### Chemicals

All chemicals were purchased from Sigma Chemical Co. (USA), except for IL-10 and TGF-β ELISA kits (eBioscience, USA). Ghrelin (Innovagen, Sweden) was dissolved in saline. Formalin solution was prepared by diluting 37% formaldehyde stock solutions in saline to obtain 5% formalin. Solutions were freshly prepared on the day of experiment by dissolving in normal saline (0.9% NaCl).

### Animals

Adult male Wistar rats (220-250 g) used in this study were obtained from Laboratory Animal Unit of Tabriz University of Medical Sciences (Tabriz, Iran). Animals were kept, four per cage, under 12:12 light/dark schedule and at a temperature of 25±2ºC with unlimited access to food and water. All experiments were performed under the ethical guideline of the Tabriz University of Medical Sciences for care and use of laboratory animals (National Institute of health publication No. 85-23, 1985). All experiments were carried out between 09:00 A.M. and 2:00 P.M. by an observer who was unaware of the nature of treatments, and efforts were made to reduce animal suffering and the number of animals used.

### Experimental design

Prior to the beginning of the study, the animals were randomly assigned to six groups of 8 each: control, formalin+saline, ghrelin (40, 80, and 160 μg/kg), and morphine groups. To induce formalin pain, animals received i.pl. injection of 50 μl of 5% formalin into the right hind paw. Ghrelin (40, 80, and 160 μg/kg) was administrated by i.p. route 30 min prior to formalin injection. Morphine (30 mg/kg, i.p.) was administrated 30 min before formalin injection. The control rats received normal saline by i.p. route. All i.p. injections were performed in a constant volume of 10 ml/kg.

### Formalin test

For the assessment of formalin-induced inflammatory nociception, a plexiglas observation chamber (30 cm in diameter and in height) was used. A mirror positioned at a 45° angel under the floor for monitoring animal behavior without moving the chamber. Rats were placed individually inside the chamber at least 15 min to acclimatize to handling and testing before being individually tested. In formalin (50 µl, 5%) groups, following the injection, rats were immediately returned to the observation chamber, and the nociception behavior was videotaped for 30 min.

In the formalin test, nociceptive response follows biphasic paradigm. It includes early or acute phase (over the initial 5 min period after formalin injection) followed by a short passive interphase and then by a late inflammatory pain phase (during the 10-15 min after formalin injection). Therefore, nociceptive behavior is assessed throughout of these two phases[15,16] and scored. The score 0 indicates normal use of the injected paw, in which plantar surface of the paw comes into full contact with the floor of the chamber; 1 shows the careful use of the injected paw, in which some parts of the paw is contacted with the floor and animal limps when walking, 2 indicates the situation in which injected paw is elevated and is not contacted with floor surface, and 3 demonstrates the vigorous shaking or licking of the injected paw[16]. Next, a nociceptive score, ranging from 0 to 3, was calculated by multiplying the time spent in each category by the category score summing these products and dividing by the total time (300 s) for each 5-min block of time[15].

[(0×T0)+(1×T1)+(2×T2)+(3×T3)]/time=score

Therefore, pain behaviors were divided into the initial early phase or the second (late) phase[16].

### Measurement of IL-10 and TGF-β serum levels

At the end of the behavioral test, blood was collected via cardiac puncture under ketamine and xylazine anesthesia (125 and 12.5 mg/kg; i.p.). Serum was then separated from blood cells by centrifugation at 1500 ×g at 4°C for 3 min and was quickly kept at -70°C until the measurements of IL-10 and TGF-β. The levels of IL-10 and TGF-β were measured using a commercially available ELISA kits (eBioscience, USA) according to the manufacturer’s instructions.

### Statistical analysis

Statistical analyses were performed using statistical package (SPSS 16 software). Data were presented as mean±SEM. Comparisons between the groups were carried out using One-way analysis of variance (ANOVA), followed by *post hoc* Tukey’s test. The significance level was set at *P*<0.05.

## RESULTS

### Effect of ghrelin on the early and late phases of inflammatory nociception

**Fig. 1:**
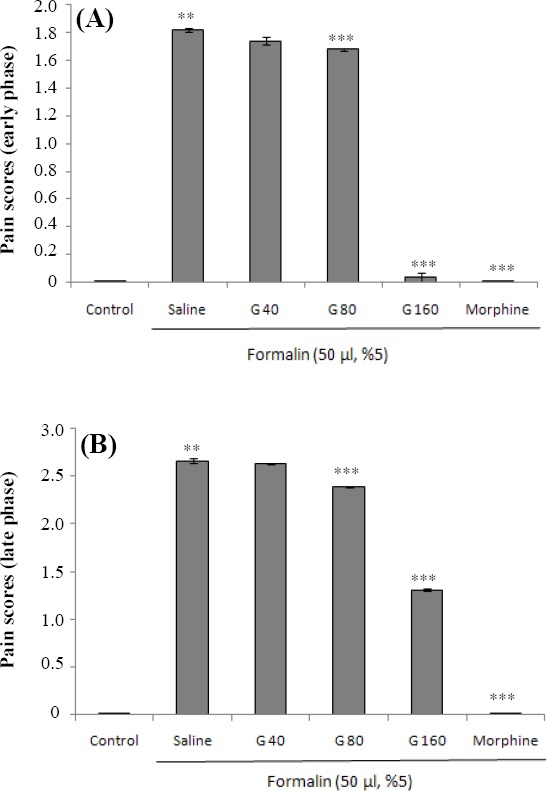
Effect of different doses of ghrelin (40, 80, and 160 µg/kg, i.p.) and morphine (30 mg/kg, i.p.) on (A) the early and (B) the late phases of pain. Each bar represents mean±SEM of pain intensity score; n=8 rats in each group; **P<0.001 when compared to control group; ***P<0.001 when compared to formalin group co-treated with normal saline. G, ghrelin

As shown in Figure 1A, i.pl. injection of formalin significantly (*P*<0.001) increased pain score in early phase in comparison with the control group. Ghrelin at the doses of 80 and 160 μg/kg, as well as the i.p. injection of morphine significantly (*P*<0.001) decreased the intensity of the early phase pain in comparison with the formalin group, indicating analgesic effect of ghrelin on the early phase of pain. Results from the late phase of pain are depicted in Figure 1B. The i.pl. injection of formalin significantly (*P*<0.001) increased pain score in the late phase when compared with the control rats. Higher doses of ghrelin (80 and 160 μg/kg, i.p.) could also significantly reduce the pain intensity (*P*<0.05 and *P*<0.001, respectively) in the late phase.

### Effect of ghrelin on serum levels of IL-10

**Fig. 2:**
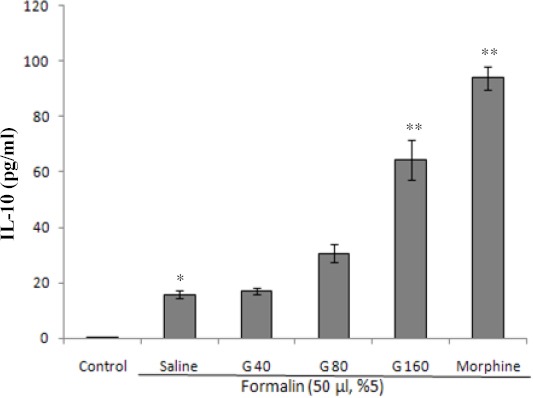
Effect of different doses of ghrelin (40, 80, and 160 µg/kg, i.p.) and morphine (30 mg/kg, i.p.) on serum levels of IL-10. Each bar represents mean±SEM of serum levels of IL-10; n=8 rats in each group; *P<0.05 when compared to control group; **P<0.001 when compared to formalin group co-treated with normal saline. G, ghrelin

Figure 2 show that the i.pl. injection of formalin remarkably elevates IL-10 serum levels when compared to the control group (*P*<0.05). Ghrelin at the doses of 80 and 160 μg/kg, as well as morphine increased the serum levels of IL-10 in comparison to the formalin+saline group. However, statistically significant increase in IL-10 with ghrelin was only observed at the dose of 160 µg/kg (*P*<0.001).

### Effect of ghrelin on serum levels of TGF-β

**Fig. 3:**
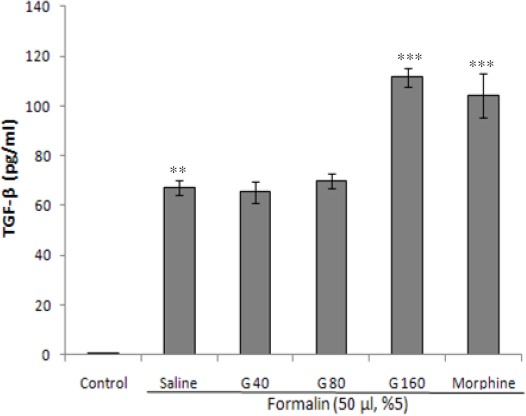
Effect of different doses of ghrelin (40, 80, and 160 µg/kg, i.p.) and morphine (30 mg/kg, i.p.) on TGF-β serum levels. Each bar represents mean±SEM of serum levels of TGF- β; n=8 rats in each group; **P<0.001 when compared to control group; ***P<0.001 when compared to formalin group co-treated with normal saline. G, ghrelin

As depicted in Figure 3, the i.pl. injection of formalin significantly raised the serum levels of TGF-β when compared to the control group (*P*<0.001). Moreover, the injection of different doses of ghrelin (40, 80, and 160 µg/kg, i.p.), as well as morphine (30 mg/kg, i.p.) augmented the TGF-β serum levels as compared with the formalin group receiving normal saline. However, realistically notable elevation in TGF-β with ghrelin treatment was detected only at the dose of 160 µg/kg (*P*<0.001).

## DISCUSSION

The present study shows that ghrelin is possessed an analgesic effect at the doses of 80 and 160 μg/kg and reduces the intensity of both the early and late phases of pain in a model of formalin test. This test is a standard and reliable model of nociception for studying compounds with probable analgesic and anti-inflammatory potentials[15]. Apparently, formalin-nociception is corresponding with expressing the different classes of cytokines. As shown by Chichorro *et al*.[17], the production of cytokine molecules such as TNF-α, IL-1β, IL-6 and IL-8 may be responsible for formalin-induced orofacial nociception in rats[17]. Moreover, it has been demonstrated that i.pl. injection of formalin stimulates the expression of TNF-α at mRNA level[18]. Taken together, these findings indicate the involvement of cytokines in the formalin-induced pain model.

In the current study, the i.pl. injection of formalin elevated the serum levels of IL-10. This observation has also been supported by the result of Shivers *et al*. study[19] who demonstrated that the i.pl. injection of formalin resulted in increased levels of IL-10 in both serum and certain brain regions such as dorsal root ganglion and spinal cord[19]. Our results also indicated that the systemic administration of ghrelin attenuates pain response both in the early and the late phases of formalin test. Moreover, the assessment of serum levels of IL-10 and TGF-β revealed the ability of ghrelin to increase these anti-inflammatory cytokines. Previous studies have shown that ghrelin induces both anti-nociceptive[12,20-22] and anti-inflammatory[11,14,20,21] effects in different pain models. These effects are possibly mediated through growth hormone secretagogue receptor-1α and central opioidergic system[11,20]. Ghrelin has a positive impact on opioid peptide release. Several studies have reported that ghrelin interacts with endogenous opioid system through the activation of proopiomelanocortin neurons, which play an important role in analgesia[11,12,23]. Furthermore, Santos *et al*.[24] showed that the i.p. injection of formalin recruits lymphocytes and neutrophils to the site of injection. T regulatory cells have ability to produce the high levels of IL-10 and TGF-β[25]. Moreover, T cells express the mRNA of ghrelin and ghrelin receptor[26]. Ghrelin is able to inhibit the expression of the pro-inflammatory cytokines such as IL-1β, IL-6, and TNF-α, which are involved in the development of inflammatory and neuropathic pain[7,11,14,21,27]. Moreover, ghrelin has the potential to increase anti-inflammatory cytokine IL-10 from lipopolysaccharides -stimulated macrophages[28]. Therefore, the analgesic effect of ghrelin in this study in both early and late phases is possibly through the release of anti-inflammatory cytokines or the inhibition of pro-inflammatory cytokines release by immune cells in the site of inflammation.

In addition, the elevation of both IL-10 and TGF-β inhibits the production of the pro-inflammatory cytokines such as TNF-α, IL-1α, and IL-1β[29]. Previous studies have shown that the systemic and intra-cerebral administration of pro-inflammatory cytokine in rats can produce hyperalgesia, and silencing these cytokines results in the elimination of their anti-analgesic effects[29-31]. It has also been indicated that TGF-β1 treatment represses nerve injury-induced inflammatory response in the spinal cord through decreasing the cytokine expression level[32]. TGF-β has also been shown to attenuate neuropathic pain via the inhibition of T lymphocytes and macrophages infiltration[33]. Moreover, in the central nervous system, TGF-β1 alleviates neuropathic pain via different mechanisms such as the modulation of neuron-glia interaction and the spinal cord expression of endogenous opioids[34].

The findings of our study demonstrated that morphine increases the serum levels of IL-10 and TGF-β. These findings have also been supported by the study of Schwartz *et al*.[35] who observed that morphine increases accumbal IL-10 gene expression in neonatal rat. It has also been demonstrated that morphine has highly stimulatory effect on the release of anti-inflammatory cytokines, such as TGF-β1 and IL-10, from the isolated spleenocyte of mice[36].

The results of the present study showed that ghrelin reduced both the early and the late phases of inflammatory pain, possibly through an increase in the serum levels of IL-10 and TGF-β, as anti-inflammatory cytokines. Ghrelin may thus offer a new alternative for the treatment of pain in inflammatory states.
